# Musculocutaneous Nerve Compression by a Humeral Osteophyte: An Unusual and Difficult-to-Diagnose Cause of Peripheral Neuropathy

**DOI:** 10.7759/cureus.93453

**Published:** 2025-09-28

**Authors:** Romain Mari, Guillaume Huttin

**Affiliations:** 1 Department of Reconstructive Surgery, Hand Surgery Unit, Université Grenoble Alpes, Centre Hospitalier Universitaire de Grenoble Alpes, Grenoble, FRA

**Keywords:** musculocutaneous nerve, nerve entrapment, osteophyte, peripheral neuropathy, upper limb

## Abstract

Isolated neuropathy of the musculocutaneous nerve is an uncommon condition, and compression by a bony outgrowth represents an exceptionally rare cause. We describe the case of a 58-year-old man who presented with motor and sensory deficits consistent with musculocutaneous nerve dysfunction. Cross-sectional imaging and nerve conduction studies revealed a proximal humeral osteophyte responsible for nerve compression. The patient underwent surgical excision of the osteophyte, which resulted in a favorable postoperative outcome. This case highlights the rarity of osteophyte-related nerve compression and underscores the importance of considering an osseous etiology in the differential diagnosis of atypical peripheral neuropathies. Imaging modalities such as CT and MRI, in combination with electrophysiological testing, play a central role in lesion identification. Surgical management offers the potential for meaningful functional recovery.

## Introduction

Peripheral neuropathies of the upper limb are relatively common, yet isolated involvement of the musculocutaneous nerve remains an uncommon entity [1]. The musculocutaneous nerve, originating from the lateral cord of the brachial plexus (C5-C7), provides motor innervation to the coracobrachialis, biceps brachii, and brachialis muscles, while also ensuring sensory supply to the lateral aspect of the forearm via the lateral antebrachial cutaneous nerve. Given its anatomical course, particularly its passage through the coracobrachialis muscle, it is vulnerable to certain entrapment or compression syndromes [2,3].

No population-based data exist for the overall incidence of isolated musculocutaneous neuropathy. In a large electromyography cohort over 18 years, only 32 cases were identified, highlighting the exceptional rarity of this condition [1]. The causes of musculocutaneous neuropathy reported in the literature include penetrating or blunt trauma, iatrogenic injury during surgical procedures, and congenital or acquired anatomical variations [1,4,5]. In contrast, compressive neuropathies due to osseous structures such as osteophytes are exceedingly rare. Due to their atypical presentation, these cases may be misdiagnosed as radiculopathies or other peripheral nerve lesions, resulting in delays in appropriate management.

We present a case of musculocutaneous nerve compression caused by a humeral osteophyte, illustrating both the diagnostic challenges and the therapeutic considerations. The study aims to discuss the clinical features, the role of imaging and electrophysiological studies, and the importance of surgical management in achieving functional recovery.

## Case presentation

A 58-year-old man with no significant past medical history presented with progressive right upper limb pain over the preceding three months. He had initially been followed for two years for atypical neurological symptoms of the right upper limb, which had been misdiagnosed as a right C6-C7 radiculopathy prior to referral.

The patient reported a recent loss of strength over the past three months, associated with atrophy of the biceps brachii muscle. On clinical examination, there was reduced strength in the biceps brachii and brachialis muscles, marked hypoesthesia along the lateral forearm, and an absent biceps reflex. Atrophy of the biceps brachii was evident. No additional motor or sensory deficits were noted in other nerve territories of the upper limb.

**Figure 1:**
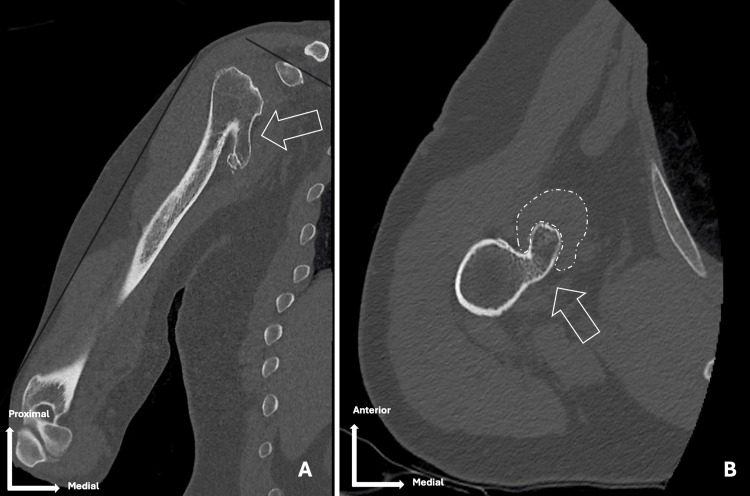
(A) Coronal and (B) axial view CT scan (bone window) White arrows indicate a large proximal humeral osteophyte penetrating the coracobrachialis muscle (white dotted line) near the musculocutaneous nerve CT: computed tomography

A CT scan revealed a large osteophyte of the right humerus, located medially at the humeral neck, measuring approximately 5 cm along its greatest axis, in direct relation to the course of the musculocutaneous nerve (Figure 1).

**Figure 2:**
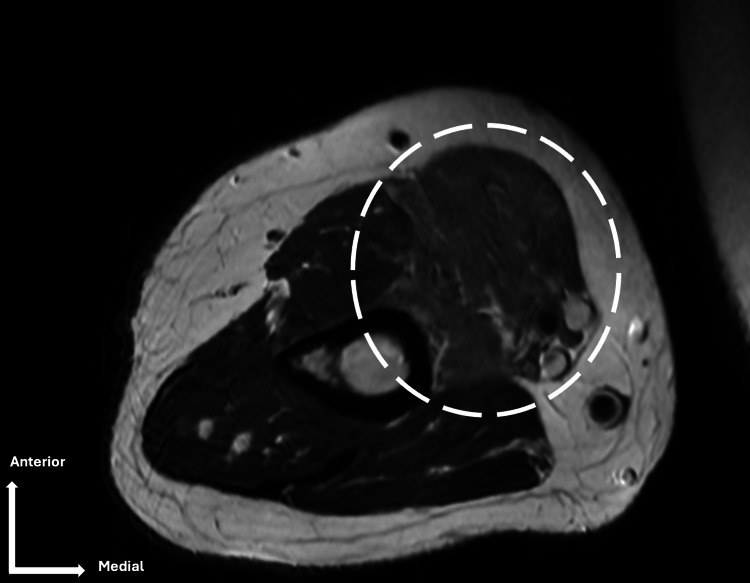
Axial MRI (T2) Hypersignal in the biceps brachii muscle (white circle). MRI: magnetic resonance imaging

MRI demonstrated signal abnormalities in the biceps brachii muscle, consistent with musculocutaneous neuropathy (Figure 2).

Electromyography, performed as a needle EMG only, demonstrated denervation potentials confined to the biceps brachii muscle; no nerve conduction study of the right musculocutaneous nerve was performed.

The patient underwent surgical excision of the osteophyte with concomitant neurolysis of the musculocutaneous nerve, resulting in rapid pain relief. Postoperative histological analysis confirmed the diagnosis of an osteophyte, characterized by a cartilaginous cap with enchondral ossification and underlying lamellar bone formation, thereby confirming the diagnosis and ruling out evidence of malignancy. At the six-month follow-up, partial recovery of biceps brachii muscle strength was observed (from 3/5 preoperatively to 4/5 postoperatively), along with significant improvement in sensory deficits. The patient was fully informed that data and images from the case would be submitted for publication and gave his verbal consent.

## Discussion

Isolated neuropathy of the musculocutaneous nerve is a rare entity, with reported etiologies most often related to trauma, iatrogenic injury, or anatomical variations [1,4,5]. The diagnosis can be particularly challenging. In our case, the patient had been monitored for two years for atypical neurological symptoms of the upper limb, initially misdiagnosed as a C6-C7 radiculopathy. The sudden worsening of symptoms prompted reconsideration of the original diagnosis.

Anatomically, the musculocutaneous nerve pierces the coracobrachialis muscle, then courses between the biceps brachii and brachialis muscles, providing motor innervation to both. Distally, it continues as the lateral antebrachial cutaneous nerve, which supplies sensation to the anterolateral forearm. Two potential compression sites can therefore be identified: within the coracobrachialis, producing combined motor and sensory deficits [6], and at the elbow, where the lateral antebrachial cutaneous nerve may be compressed beneath the biceps aponeurosis and brachialis fascia, leading to isolated sensory involvement [7].

In our case, elbow flexion remained largely preserved due to compensatory activation of the epicondylar muscles, allowing flexion with the forearm pronated, a mechanism known as the “Steindler effect” [8].

Osteophytes may result from joint degeneration, repetitive microtrauma, prior fractures, or systemic conditions. In our case, no such factors were identified, suggesting that the humeral osteophyte was idiopathic. Mechanical compression by a bony formation is exceptional. To date, the literature reports only two comparable cases: one of musculocutaneous neuropathy secondary to a humeral osteophyte [9] and another due to a humeral osteochondroma [10]. Our observation adds to these rare descriptions and underscores the importance of considering an osseous compressive origin when faced with atypical peripheral neuropathy.

More broadly, osteophyte-related nerve compressions have been described in other locations, including the median nerve [11], confirming that although rare, osteophytes can give rise to diverse and sometimes misleading neurological presentations. Imaging and electromyography remain essential tools for identifying the site of compression and guiding appropriate management.

Surgery appears to be the treatment of choice in compressive etiologies, with most reported cases achieving satisfactory functional recovery [9,10]. The favorable postoperative outcome in our patient following osteophyte excision further illustrates the effectiveness of this approach.

## Conclusions

Isolated neuropathy of the musculocutaneous nerve remains a diagnostic challenge owing to its rarity and the overlap of clinical manifestations with cervical radiculopathies or lesions involving adjacent peripheral nerves. This case underscores the importance of considering an osseous etiology when evaluating atypical neurological symptoms of the upper limb. Advanced imaging modalities, such as CT and MRI, in conjunction with electromyography, are crucial for establishing a diagnosis and elucidating the underlying pathology. Surgical management through osteophyte excision and nerve decompression constitutes the treatment of choice, frequently leading to pain relief and functional recovery. This report contributes to the limited body of literature on osteophyte-induced musculocutaneous neuropathy. It reinforces the importance of a multidisciplinary approach involving neurologists, radiologists, and peripheral nerve surgeons to optimize patient outcomes.
